# N-Graphene Nanowalls via Plasma Nitrogen Incorporation and Substitution: The Experimental Evidence

**DOI:** 10.1007/s40820-020-0395-5

**Published:** 2020-02-17

**Authors:** Neelakandan M. Santhosh, Gregor Filipič, Eva Kovacevic, Andrea Jagodar, Johannes Berndt, Thomas Strunskus, Hiroki Kondo, Masaru Hori, Elena Tatarova, Uroš Cvelbar

**Affiliations:** 1 Jožef Stefan Institute, Jamova cesta 39, 1000 Ljubljana, Slovenia; 2 Jožef Stefan International Postgraduate School, Jamova cesta 39, 1000 Ljubljana, Slovenia; 3 GREMI CNRS-University of Orleans, 14 rue d’Issoudun, 45067 Orleans Cedex 2, France; 4 Institute for Materials Science, Christian Albrechts University Kiel, Kaiserstr, 2, 24143 Kiel, Germany; 5 Department of Electrical Engineering and Computer Science, University of Nagoya, Furo-cho Chikusa-ku, Nagoya, Aichi 464-8603 Japan; 6 Instituto de Plasmas e Fusão Nuclear, Instituto Superior Técnico, Universidade de Lisboa, 1049 Lisbon, Portugal

**Keywords:** Graphene, Graphene nanowalls, Plasma post-treatment, Nitrogen incorporation, Raman spectroscopy, Vacancy defects

## Abstract

Incorporating nitrogen (N) atom in graphene is considered a key technique for tuning its electrical properties. However, this is still a great challenge, and it is unclear how to build N-graphene with desired nitrogen configurations. There is a lack of experimental evidence to explain the influence and mechanism of structural defects for nitrogen incorporation into graphene compared to the derived DFT theories. Herein, this gap is bridged through a systematic study of different nitrogen-containing gaseous plasma post-treatments on graphene nanowalls (CNWs) to produce N-CNWs with incorporated and substituted nitrogen. The structural and morphological analyses describe a remarkable difference in the plasma–surface interaction, nitrogen concentration and nitrogen incorporation mechanism in CNWs by using different nitrogen-containing plasma. Electrical conductivity measurements revealed that the conductivity of the N-graphene is strongly influenced by the position and concentration of C–N bonding configurations. These findings open up a new pathway for the synthesis of N-graphene using plasma post-treatment to control the concentration and configuration of incorporated nitrogen for application-specific properties.

**Electronic supplementary material:**

The online version of this article (10.1007/s40820-020-0395-5) contains supplementary material, which is available to authorized users.

## Highlights

- Nitrogen was successfully incorporated in graphene nanowalls (CNWs) using cold gaseous plasma post-treatment and influence of nitrogen concentration and configuration in CNWs on electrical conductivity was demonstrated.
- The mechanism of nitrogen incorporation was systematically studied using different characterisation techniques to make a bridge between established DFT theories.

**Electronic supplementary material:**

The online version of this article (10.1007/s40820-020-0395-5) contains supplementary material, which is available to authorized users.

## Graphical abstract

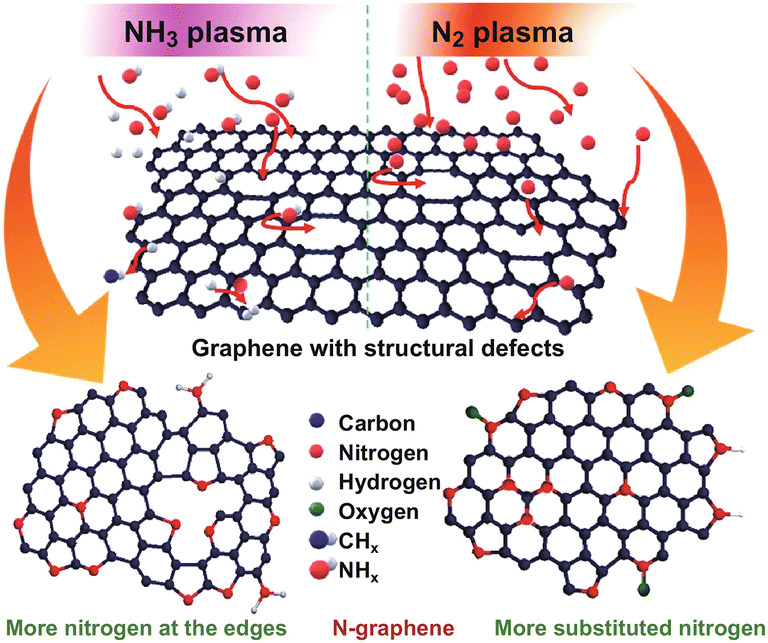

## Introduction

Carbon nanowalls (CNWs) or graphene nanowalls are considered as two-dimensional (2D) graphite sheet nanostructures composed of stacks of graphene sheets with open boundary edges standing vertically on the substrate and widely synthesised using the plasma-enhanced chemical vapour deposition [1–5]. The nucleation and growth of nanosized structures on the substrate are influenced by temperature, plasma power and pressure, which are controlling the thickness of self-aligned nanowalls in the range of few nanometres to a few tens of nanometres with an interlayer spacing of few nanometres [6–9]. Catalyst-free synthesis, large surface area, sharp edges with open boundaries and ease of functionalisation of CNWs can be suitable for numerous applications such as field emission [10, 11], electrochemical and energy storage applications [12–18] and novel electronic devices [19, 20]. Thus, tailoring the electrical characteristics of CNWs/nanocarbons for achieving application-specific properties has attracted wide attention, specifically by incorporating a heterogeneous atom for controlling the surface properties [21], tuning the bandgap [22] and tailoring the electrical properties [23–25].

**Fig. 1:**
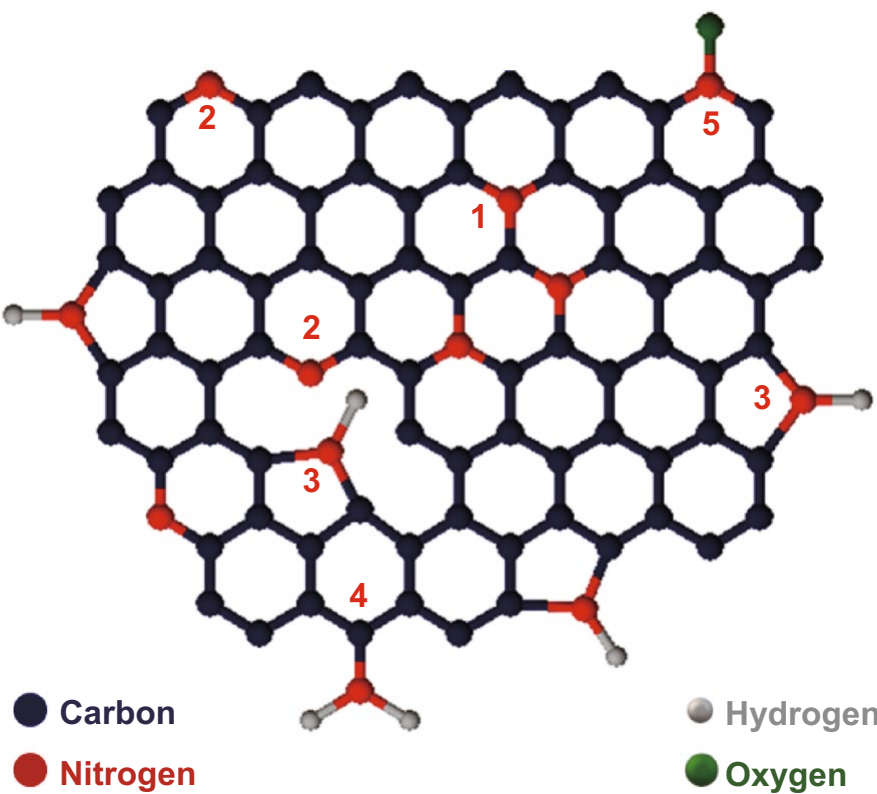
Possible nitrogen configurations in graphene lattice, labelled as (1) graphitic N, (2) pyridinic N, (3) pyrrolic N, (4) amine, and (5) N-oxides of pyridinic N

Among all the heterogeneous atom incorporation mechanisms, incorporation of nitrogen in the CNWs/graphene has potential research interests because of its ability to tune and modify the electrical properties of CNWs. Fabrication of nitrogen-incorporated CNWs (N-CNWs) can be done either by in situ method or by a post-treatment [26, 27]. In situ synthesis of N-CNWs occurs during the growth of CNWs, while in the post-treatment method, the pre-prepared CNWs are treated in a nitrogen-containing atmosphere to produce N-CNWs with homogenous incorporation of nitrogen. Post-treatment of CNWs with nitrogen precursors has been widely used for incorporating nitrogen on the surface of graphene-like structures with controlled nitrogen bonding, which is mainly done with thermal and plasma post-treatments  [28]. Incorporated nitrogen concentration is low in the thermal treatment method due to the insufficient defect number in the graphene lattice and high annealing temperature [28–30]. Compared to the thermal treatment, when carbon materials are exposed to a nitrogen plasma atmosphere, carbon atoms will partly be replaced by the N-atoms and N-graphene with higher nitrogen concentration will be produced. This is mainly due to the creation of the excited nitrogen species (either nitrogen-containing molecules, or atoms), reactive N-atoms or even ions and the defect generation on the graphene surface during plasma processing. These reactive species can influence the structure of CNWs through the crystallinity of the structure and nitrogen configurations or concentrations by controlling the plasma conditions and exposure time [26, 31–33]. Based on the position of incorporated nitrogen, the possible configurations of nitrogen in N-graphene such as pyridinic N (N-6, 398.1–399.3 eV), pyrrolic N (N-5, 399.8–401.2 eV), graphitic N/quaternary N (N–Q, 401.1–402.7 eV), amines (399.0–399.7 eV) and N-oxides of pyridinic–N (N–O, 403–405 eV) are presented in Fig. 1 [30, 34].

Pyridinic N bonds to two carbon atoms at the graphene edges and contributes one p-electron to the π system [33], and is weakly bonded to the graphene lattice due to the existence of dangling bonds [35]. Pyrrolic N has a five-membered ring structure, which contributes two p-electrons to the π system [36]. The N-atom substitutes one of the missing carbon atoms in the hexagonal ring to form graphitic N. Among these configurations, pyridinic N and graphitic N are *sp*^2^-hybridised [37], and pyrrolic N is *sp*^3^-hybridised [38]. Apart from these three common nitrogen configurations, amines have been observed at the carbon atoms situated at the edges and oxidised groups of N observed at pyridinic N. High content of pyridinic N and pyrrolic N reduces the conductivity due to the existence of a large number of nanoholes and *p*-type doping [39]. Thus, graphitic N can be considered as the most promising nitrogen configuration for tuning the electrical properties due to the strong *n*-type doping effect without any significant defect generation [40].

Manipulation of structural defects is an effective approach for the intrinsic modification of graphene. Stone–Wales (SW), single or double vacancies (SV and DV), nanoholes and the edges of graphene are the active defect sites for the incorporation of a heterogeneous atom [41, 42]. Transformation of the four neighbouring hexagonal rings into two pentagons and two heptagons by the rotation of one of the carbon bonds causes SW defect. SV defect is formed due to the missing of a carbon atom in the hexagonal ring, which forms a five-membered and nine-membered ring structure known as “SV(5-9)”. DV can be formed either by the coalescence of two SV or by the removal of two neighbouring carbon atoms. The latter forms an energetically unstable structure with two pentagons and one octagon known as “DV (5-8-5)”. Thus, typically, one of the carbon atoms in “DV (5-8-5)” rotates for 90° and forms “DV (555-777)” with a ring structure having three pentagons and heptagons. These defects can be formed either during the growth of graphene-like structures or during the intrinsic modification. The interaction between these defects and heterogeneous atoms leads to the incorporation of nitrogen with different configurations.

Recent studies on the synthesis of N-CNWs using nitrogen-containing plasma treatment suggested that the structural defects are responsible for the formation of N-graphene with different N configurations [16, 43]. The reported DFT calculations based on different experimental conditions such as plasma exposure [16], thermal annealing [37, 44] and nitrogen ion implantation [45, 46] suggested that SV and DV (555–777) defects are the most favourable sites for the nitrogen incorporation, and SW and DV (5-8-5) defects are unlikely to trap N-atoms to form N-graphene [16]. Depending on the cooperative effect between the dopant and defects, pyridinic N in graphene lattice can be formed with an SV [45, 47], and pyrrolic N can be formed along with pyridinic N at DV [47]. Embedding of excited N-atom in the carbon vacancy and migration of carbon atom in the presence of a C-adatom at an SV is the possible mechanism to form graphitic N by substituting the missing C-atom using an N-atom, and this restores the hexagonal ring [16, 45, 48]. All these DFT calculations suggest that the interactions of nitrogen with the defect sites are the key to the efficient building of N-graphene. Here, one of the main challenges lies in the synthesis of N-CNWs/N-graphene with desired nitrogen configurations. However, there is a lack of experimental results to explain the mechanism of nitrogen incorporation in the structural defects of graphene and compare evidence with the proposed DFT theories.

Conjugating Near Edge X-Ray Absorption Fine Structure (NEXAFS) spectroscopy with different characterisation techniques opens up the possibility to extract most of the chemical information of the graphene such as coordination environment, geometrical distortions and different defect types in graphene [49, 50]. To date, different groups confirmed theoretically that the formation of additional peaks in the “fingerprint” region (binding energy ~ 286–290 eV) of the graphene NEXAFS spectrum is mainly due to the structural defects [49, 50]. Significant variations in the NEXAFS “fingerprint” region due to the interaction of heterogeneous atoms with the structural defects are also reported elsewhere [51, 52]. Thus, comparing various established DFT calculations of the NEXAFS spectra with experimental results can enlighten the mechanism of interaction of nitrogen with the structural defects to form N-CNWs.

In this work, we demonstrate the effect of different nitrogen-containing plasma post-treatments for the incorporation of nitrogen into CNWs using ammonia (NH_3_) and nitrogen (N_2_) in an inductively coupled RF low-temperature plasma. Furthermore, we present a systematic study on the effect of plasma exposure time on the structure and morphology of CNWs using Raman spectroscopy and scanning electron microscopy (SEM). The time-dependent changes in the nitrogen configurations and concentrations are evaluated by X-ray photoelectron spectroscopy (XPS). NEXAFS spectroscopy is used to distinguish the structural changes and defect generation occurring during the plasma exposure time. Van der Pauw method is used to measure the electrical conductivity of the CNWs and N-CNWs and to understand the effect of nitrogen configuration on electrical conductivity. Finally, the obtained results are used to explain the effect of the plasma environment on the creation of defects in graphene and to unravel the mechanism of nitrogen incorporation into graphene when forming N-CNWs.

## Experimental Section

### Synthesis of N-CNWs

The CNWs were synthesised on a SiO_2_ substrate using a double plasma device for CNWs deposition. The system consisted of a surface wave plasma (SWP) region driven by a 2.45 GHz microwave power supply to generate H-atoms and a capacitively coupled plasma (CCP) region generated by a 100 MHz power supply to fabricate nanowalls from CH_4_ gas. The high-density H-atoms were generated at the top of the PECVD system by the SWP system, which was operated at 2.54 GHz and injected into the PECVD system. The CNWs were then grown on the substrate. The details are described elsewhere [53–55]. For the nitrogen doping and synthesis of N-CNWs, plasma post-treatment was carried out in an RFICP (radio-frequency inductively coupled plasma) system that consists of an 80-cm-long glass tube with a diameter of 4 cm. The RF generator was inductively coupled to the system by using a nine-turn water-cooled inductive coil. Ammonia (NH_3_) and nitrogen (N_2_) gases were used as the dopant gases at the flow rate of 100 sccm. The total pressure in the chamber before experiments was below 1 Pa, and during the experiments, it was not above 30 Pa. The sample was placed 10 cm away from the centre of glow discharge into the post-glow region. This was done in order to have low-temperature treatments of samples, where the surface temperature was never above 50 °C, typically even much less. Post-glow region also contained mostly the high densities of neutral reactive species and the negligible densities of ions. Plasma was generated at an RF power of 300 W. The plasma post-treatment was carried out in an incremental method, where the plasma was switched off during each treatment periodically to keep the surface temperature minimised. This was done typically in different steps of 4, 5 or 10 s and equal cooling periods. Depending on the aggressiveness of used nitrogen gases towards the samples and its consequent etching, the most optimal time treatments were selected. For the CNWs, the typical samples treated with NH_3_ plasma in the post-glow region were 4, 8, 12 and 25 s with an incremental order of 4 or 5 s, and for N_2_ plasma 10, 20, 30 and 40 s with an incremental order of 10 s. The schematic diagram of the posttreatment experimental setup is presented in Fig. S1.

### Characterisation Techniques

The SEM characterisation of the samples was performed by a JEOL JSM-7600F, field emission scanning electron microscope using 5 kV electron acceleration voltage. The samples were placed on a double-sided carbon tape mounted on an aluminium stub. Raman spectra were recorded at different regions of the samples to study the structural properties of the CNWs and N-CNWs using NTEGRA confocal Raman spectrometer at an excitation wavelength of 633 nm with an incident power ~ 3 mW at a spot size of 50 µm. X-ray photoelectron spectroscopy (XPS) analyses were carried out on the PHI-TFA XPS spectrometer produced by Physical Electronics Inc. and equipped with Al monochromatic source emitting photons at the energy of 1486.6 eV. The analysed area was 0.4 mm in diameter with a takeoff angle (TOA) of 45°. The high-energy-resolution spectra were acquired with energy analyser operating at a resolution of 0.6 eV and a pass energy of 29 eV. The accuracy of binding energies was about ± 0.3 eV. Quantification of surface composition was performed from XPS peak intensities taking into account relative sensitivity factors provided by the instrument manufacturer [56]. The XPS spectra were analysed using MultiPak software, and Gauss–Lorentz method was used for the deconvolution of the peaks considering Shirley-type background deduction. Near Edge X-Ray Absorption Fine Structure (NEXAFS) spectroscopy was performed at Helmholtz-Zentrum Berlin (BESSY II), at High Energy Spherical Grating Monochromator (HESGM) beam line in an ultrahigh vacuum chamber (PREVAC end station). NEXAFS measurements were performed at different incident angles (20°, 30°, 45°, 55°, 70° and 90°) relative to the substrate surface. NEXAFS data on the C K edge were obtained in the total electron yield mode (TEY) with an energy resolution of ≈ 0.40 eV, 0.55 eV at the N K edge and 0.6 eV at O K edge using a home-built double-channel plate detector [57–59]. This mode enables a probing depth of about 10 nm. The further step was measurements in partial electron yield mode obtained at − 150 V (C K edge) or − 250 V (N K edge), giving information on the very surface of the material. All spectra were referenced against a peak at 284.9 eV in simultaneously recorded *I*_0_ spectra of a carbon-contaminated Au grid, which was previously referenced against the π* resonance of highly oriented pyrolytic graphite at 285.38 eV. The raw NEXAFS spectra were normalised to the incident photon flux and corrected for the beamline transmission by division through a spectrum of a clean, freshly sputtered Au sample. Finally, the C K-edge spectra have been normalised to 1 at the energy of 325 eV. Van der Pauw method was used to measure the electrical conductivity of the samples. The measurements were done by using a TTIQL564P power supply as the current source, TENMA 72-7732A multimeter as the ammeter and an Agilent/Keysight 34970A data acquisition unit as the voltmeter.

## Results and Discussion

**Fig. 2:**
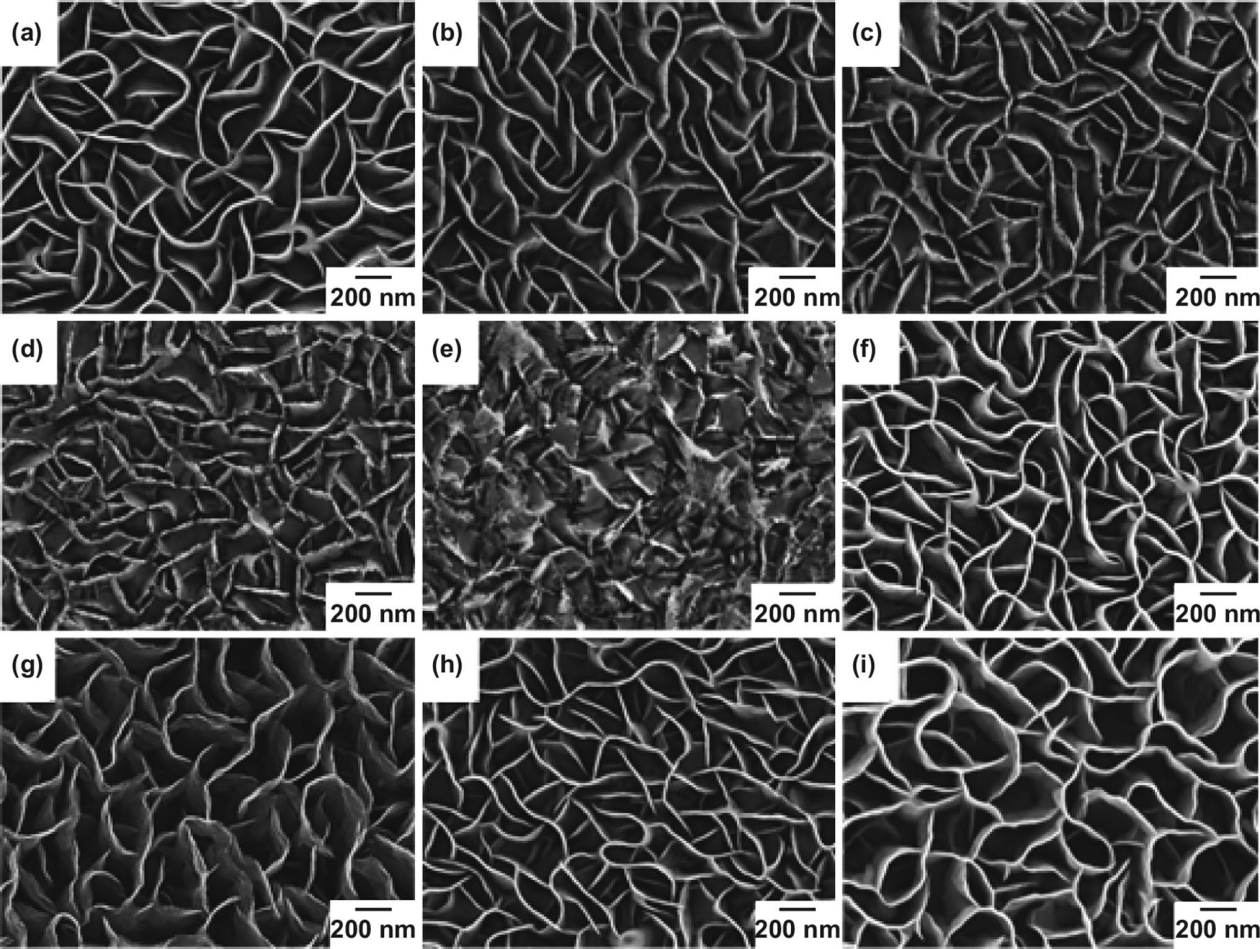
SEM images of **a** nontreated CNWs, **b**–**e** 10 s, 20 s, 30 s, and 40 s of N_2_ plasma-treated CNWs, **f**–**i** 4 s, 8 s, 12 s, and 25 s of NH_3_ plasma-treated CNWs

The morphology of CNWs in Fig. 2a exhibits the uniform growth and characteristic morphology of the as-grown CNWs. The height of CNWs was about 600 ± 100 nm, with a thickness of approximately 10 nm (Fig. S2). The typical morphological characteristics of 10, 20, 30 and 40 s N_2_ plasma-treated CNWs are presented in Fig. 2b–e. There was no noticeable change after the initial 10 s treatment. Another 10 s plasma treatment leads to the increase in roughness of nanowalls due to the etching of carbon atoms by plasma. The SEM images after 30 s and 40 s indicate that etching of nanowall structures increased with the plasma exposure time. Figure 2f–i exhibits the morphology of NH_3_ plasma-treated CNWs. Compared to the N_2_ plasma-treated samples, the morphology of NH_3_ plasma-treated samples was not significantly changed during the plasma exposure. There were no observable changes in the edges of the nanowall or structure even after 25 s plasma treatment. This effect is due to the low concentration of incorporated nitrogen compared to N_2_ plasma post-treatment. However, here it is worth noticing that also treatment periods were shortened due to the aggressiveness of NH_3_ plasma.

**Fig. 3:**
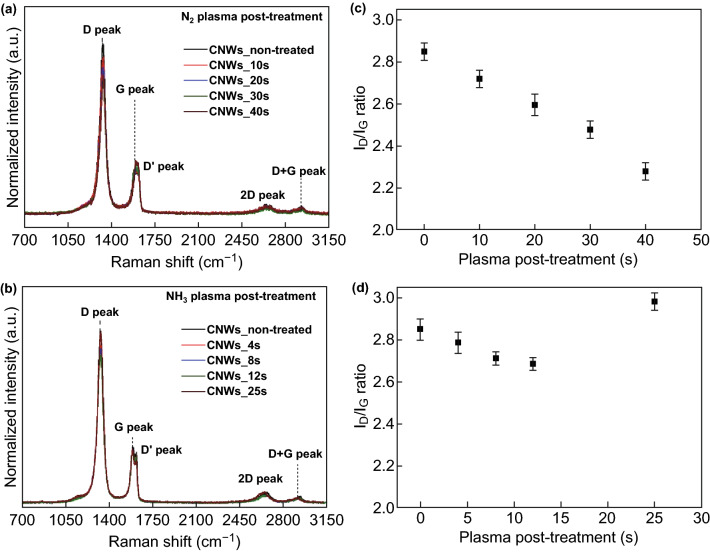
**a**, **b** Raman spectra of CNWs before and after N_2_ and NH_3_ plasma post-treatments. **c**, **d** *I*_D_/*I*_G_ ratio of CNWs before and after N_2_ and NH_3_ plasma post-treatments

Raman spectroscopy was further used to understand the structure and quality of graphene nanowalls. Typical Raman spectra of CNWs before and after N_2_ and NH_3_ plasma post-treatments are presented in Fig. 3a, b. The G-band peak is the first-order Raman band of the all *sp*^2^-hybridised carbon material, which corresponds to the six-membered ring structure of the graphene. The D band is the defect-activated band in the *sp*^2^-hybridised carbon and corresponds to the imperfection in the graphene lattice. There is a shoulder peak observed along with G peak, known as D’ peak, which is also a defect peak mainly due to the localised vibrational modes of randomly distributed impurities in the graphitic edges. The samples exhibit bands in the higher shift ranges (> 2400 cm^−1^), which are 2D (G’) band and D + G band. The presence of the D band and a very weak 2D peak is the characteristic of multilayered graphene with a number of structural defects. The peak positions for all samples are almost similar and possess the main characteristic peaks of graphene [60]; D peak is observed in between 1330 and 1333 cm^−1^, G peak at 1584–1587 cm^−1^, D’ peak at 1612–1618 cm^−1^, 2D peak at 2360–2365 cm^−1^ and D + G peak at 2917–2919 cm^−1^. The change in intensities of D and G peaks after plasma treatment is represented in Fig. S3. The quantitative analysis technique to estimate the degree of disorder in the graphene lattice is the ratio between the intensity of D and G bands (*I*_D_/*I*_G_) [61].

The evaluation of *I*_D_/*I*_G_ ratios of CNWs before and after N_2_ and NH_3_ plasma treatments is delineated in Fig. 3c, d. It is seen that the *I*_D_/*I*_G_ ratio decreases to a certain minimum as the post-treatment time is increased. The *I*_D_/*I*_G_ ratio of as-grown CNWs is 2.85, which decreases with N_2_ plasma treatment and reaches a minimum of 2.28 after the 40 s of post-treatment. Compared to N_2_ plasma, the *I*_D_/*I*_G_ ratio of NH_3_ plasma-treated CNWs exhibits very small changes with the initial plasma exposure and attains a minimum of 2.68 after 12 s treatment. Nevertheless, the prolonged plasma treatment for 25 s causes the increase of the *I*_D_/*I*_G_ ratio to 3, which is higher than the initial nanowall condition. From the *I*_D_/*I*_G_ ratio, in-plane crystallite size (*L*_a_) of CNWs was calculated using the Tuinstra–Koenig relationship [62] and is presented in Table S1. The value of *L*_a_ for as-grown CNW is 13.53 nm, which increases to 16.90 nm after the 40 s of N_2_ plasma treatment. On the other hand, for NH_3_ plasma-treated CNWs, the highest *L*_a_ value of 14.36 nm was obtained after 12 s and a minimum of 12.93 nm was attained after 25 s. These *L*_a_ values after NH_3_ plasma treatment do not have significant deviations from the initial *L*_a_ value (13.53 nm) of the CNWs. Considering *L*_a_ as average interdefects distance, an increase in *L*_a_ value indicates a lower defect concentration in the graphene lattice [30]. Thus, the defect sites were lowered after N_2_ plasma treatment either by the substitution of nitrogen to the defect sites or by the etching of the amorphous carbon phase. These changes in Raman spectra illustrate that both plasma post-treatment methods can incorporate nitrogen and functionalise the surfaces without destroying the vertical alignment. However, the difference in surface morphology and the Raman behaviour of N-CNWs after different plasma exposures implies that the incorporation of nitrogen in the graphene lattice after N_2_ and NH_3_ plasma post-treatments occurs through different nitrogen bonding configurations.

**Fig. 4:**
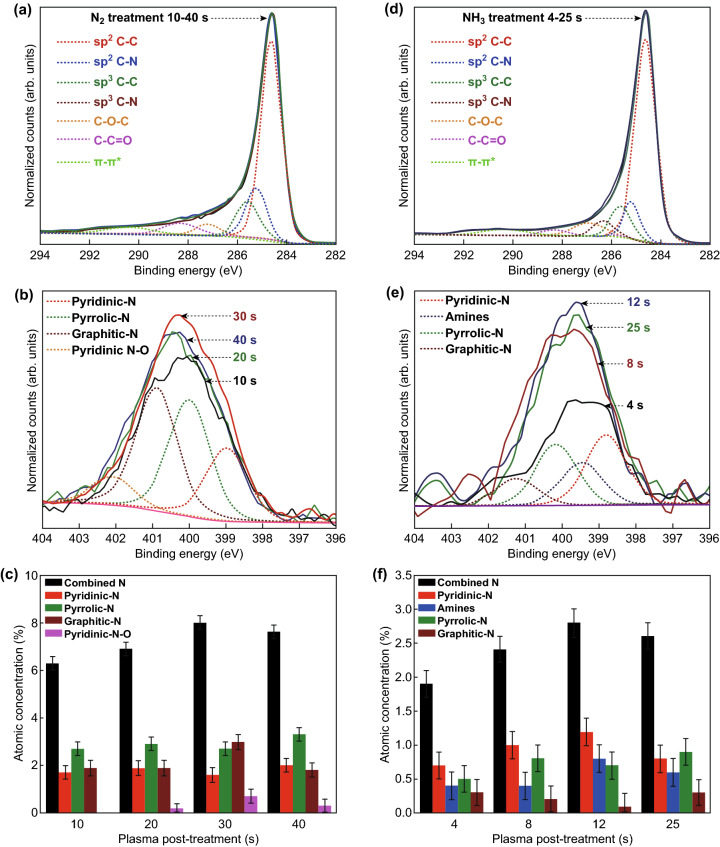
**a** High-resolution C 1s spectra, **b** N 1s spectra, and **c** content distributions of nitrogen configurations after N_2_ plasma post-treatment. **d** High-resolution C 1s spectra, **e** N 1s spectra and **f** content distributions of nitrogen configurations after NH_3_ plasma post-treatment

XPS analysis was performed to determine the elemental composition and nitrogen configurations of the N-CNWs. The nontreated CNWs had two typical peaks in the spectra: around 284.6 eV for carbon and around 532.0 eV for some residual oxygen. A peak at about 400 eV in the XPS survey spectra of all the plasma-treated samples confirms the presence of incorporated nitrogen (Fig. S4) and increase in intensity of oxygen is due to the adsorption of oxygen from the air to the reactive carbon atoms at the edges [63]. A high concentration of incorporated nitrogen (~ 8.0%) was observed after 30 s of N_2_ plasma treatment, while ~ 2.8% was the highest concentration after NH_3_ plasma treatment (Fig. S5). The change in the chemical state of carbon due to the nitrogen is a potential interest and investigated by acquiring the high-resolution XPS spectra of carbon and nitrogen. Since methane (CH_4_) was used as the precursor for the synthesis of CNWs, the H-terminated structure of nanowall also influences the bonding configurations. Figure 4a, d represents the high-resolution C 1s XPS spectra of N_2_ and NH_3_ plasma-treated CNWs. The C 1s peak after both plasma-treated CNWs is deconvoluted into different components centred at 284.6, 285.1, 285.5, 286.2, 286.9, 288.2 and 290.0 eV. All the peak positions are assigned with an uncertainty of ± 0.3 eV. The main peak of CNWs at 284.6 eV corresponds to the graphite-like *sp*^2^ C–C bond (graphitic-C) [64]. Other peaks observed at 285.1 eV are attributed to *sp*^2^ C–N, the peaks centred 285.5 eV originate from the *sp*^3^-bonded carbon atoms, peak found at 286.9 eV is ascribed to *sp*^3^ C–N bonds, carbon singly binds to oxygen at 286.9 eV and carbon in carbonyl groups at 288.2 eV. The broad peak centred at 290.0 eV represents the π–π* shake-up satellite [65]. All the samples show similar peaks after plasma treatment, and the represented deconvoluted peaks belong to the N-CNWs with higher *sp*^2^-bonded nitrogen concentration after N_2_ and NH_3_ plasma treatments. Detailed deconvolution spectra of CNWs after each plasma treatment time are presented in Fig. S6, and it is seen that the concentration of *sp*^2^ and *sp*^3^ C–N bonds of N-CNWs after N_2_ and NH_3_ plasma treatments varies with the plasma exposure time. Information of the peak positions and roughly estimated composition ratio (%) of the deconvoluted peaks of the C 1s spectrum is summarised in Tables S2 and S3.

Detailed N 1s core-level spectra were analysed to understand the evolution of different peaks of C–N configurations in C 1s spectra during the plasma post-treatment. Figure 4b, e displays the N 1s XPS spectra of N_2_ and NH_3_ plasma-treated CNWs. The N 1s peaks in all the samples were fitted into three main components, namely *sp*^2^-bonded pyridinic N (398.9 eV), *sp*^3^-bonded pyrrolic N (400.1 eV) and *sp*^2^-bonded graphitic N (401.1 eV) [40, 66]. In the case of NH_3_ plasma-treated CNWs, an additional peak is manifested at 399.5 eV, and it is ascribed to amine groups. However, in the samples treated with N_2_ plasma, a minor peak is noticed at 402.3 eV due to the oxidisation of pyridinic N by exposure to ambient air. Comparison of the deconvoluted N 1s peak intensities after different plasma and treatment time (Fig. S7) shows that the peak intensities are varying with each plasma treatment. Graphitic N and pyridinic N (*sp*^2^ C–N) components are increasing with the initial N_2_ plasma treatment. On the other hand, pyridinic N and amine components are increasing with the initial NH_3_ plasma treatment. Pyrrolic N component becomes dominant in both cases after longer plasma exposure, which is mainly because of the continuous loss of carbon atoms from graphene lattice due to the plasma etching effect. Information of the peak positions and roughly estimated concentration (%) of all the fitted peaks is presented in Tables S4 and S5.

Roughly estimated content distribution of the different nitrogen components in N-CNWs after each plasma post-treatment is represented in Fig. 4c, f. Nitrogen concentration in N_2_ plasma-treated CNWs is above ~ 6% after all the treatment conditions. Initially, pyrrolic N has a higher concentration, and the concentrations of graphitic N and pyridinic N components are increasing upon increasing plasma exposure. The higher concentration of incorporated nitrogen can be due to the formation of multiple vacancy defects by higher etching of CNWs by N_2_ plasma treatment [47]. However, the continuous loss of carbon will make the nitrogen incorporation more difficult; thus, the nitrogen concentration starts to drop and is mainly incorporated into the *sp*^3^ C–N configurations, which eventually increases the concentration of pyrrolic N. On the other hand, the nitrogen concentration is lower in the case of NH_3_ plasma-treated CNWs (Fig. 4f). The cause is likely due to the hydrogen radicals in NH_3_ plasma. Hydrogen radicals can vividly etch amorphous carbon, which can remove a significant amount of the *sp*^3^-bonded carbon from the CNWs [67, 68]. Thus, the nitrogen is incorporated into the most favourable edges of graphene in pyridinic N form, which has a higher concentration during the initial plasma exposure (4–12 s). Nevertheless, the continuous loss of carbon atom makes the nitrogen incorporation more difficult and leads to the formation of more *sp*^3^ C–N bonding and increases the concentration of pyrrolic N. The Raman studies on the in-plane crystallite sizes are strongly aligned with the XPS observations, where the crystallite size increases with the increase in *sp*^2^ C components and decreases with the increase in *sp*^3^ C concentrations. Thus, more detailed investigations are needed to reveal the effect of plasma treatment on the graphene lattice concerning the incorporation of N-atoms through a different bonding.

**Fig. 5:**
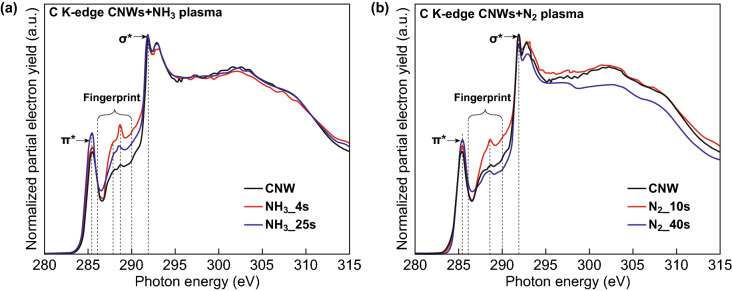
C K-edge spectra of the CNWs and N-CNWs: **a** nontreated, 4 s and 25 s NH_3_ plasma-treated CNWs, and **b** the nontreated, 10 s and 40 s N_2_ plasma-treated CNWs

NEXAFS studies were performed to gain further insight into the bonding environment of the adsorbing atom to identify the local bonding environment. Interpretation of NEXAFS signals can be used as the fingerprints to detect the sample defect fraction. C K edge spectra of the CNWs and N-CNWs after N_2_ plasma treatment and NH_3_ plasma treatment are shown in Fig. 5a, b. The spectra represented are recorded at an incident angle of 45° to avoid possible orientation effects. A characteristic sharp resonance is observed at 285.4 eV corresponding to a C 1s → π* transition. A double-structured resonance is observed at 292 eV corresponding to the C 1s → σ*, which mainly arises due to the excitonic (~ 291.7 eV) and a band-like contributions (293.1 eV) [69, 70]. The significant changes observed in the C K edge spectrum are in the region between π* and σ* resonance in the energy range between 286 and 290 eV, known as “fingerprint” region [49], attributed to the structural defects and chemical modifications in graphene [49, 71]. Thus, the evolution of the peak around 288.0 eV can be attributed to either the plasma-enhanced defect formation or incorporation of nitrogen to the defects in graphene. The experimental investigations on the NEXAFS “fingerprint” database of defects on graphene are not described well yet. Thus, the several reported DFT calculations on the NEXAFS spectra from the structural defects in graphene are used to interpret the changes in graphene lattice after plasma treatment and to explain the nitrogen incorporation mechanism [50, 52].

The model proposed by Rojas et al. [52] suggested that the substitution of a single vacancy defect with N-atom does not affect the NEXAFS signal in the “fingerprint” region, but the adsorption of N-atoms to the graphene bridge distorts it. The calculations on the NEXAFS spectra obtained from the structural defects of graphene done by Ehlert et al. [49] demonstrate that the additional peak observed at the “fingerprint” region originates from the *sp*^3^-hybridised C-atom in SV (5-9) and carbon atoms near to the defect in DV (5-8-5). Hua et al. [50] suggested that the DV has a peak with a larger intensity at the “fingerprint” region slightly above 288 eV, and SV has a peak slightly below 288 eV with lower intensity. Comparing these results with our experimental NEXAFS results confirms that the changes observed in the “fingerprint” region are mainly due to the vacancy defects. As-grown CNWs have two low-intensity peaks in the “fingerprint” region around 288.2 and 287.5 eV. It is seen that the evolution of these peaks after 4 s NH_3_ plasma treatment (Fig. 5a) and decreases the intensity with the longer plasma exposure time (25 s). Considering the above-mentioned DFT calculations on the defect effects, these peaks at 288.2 and 287.5 eV can be assigned as the characteristics of DV (5-8-5) and SV (5-9), respectively. Compared to NH_3_ plasma-treated samples, the noticeable change in the “fingerprint” region of C K-edge spectra of N_2_ plasma-treated samples is the evolution of only one peak around 288.2 eV and is the characteristic of DV (5-8-5) (Fig. 5b).

**Fig. 6:**
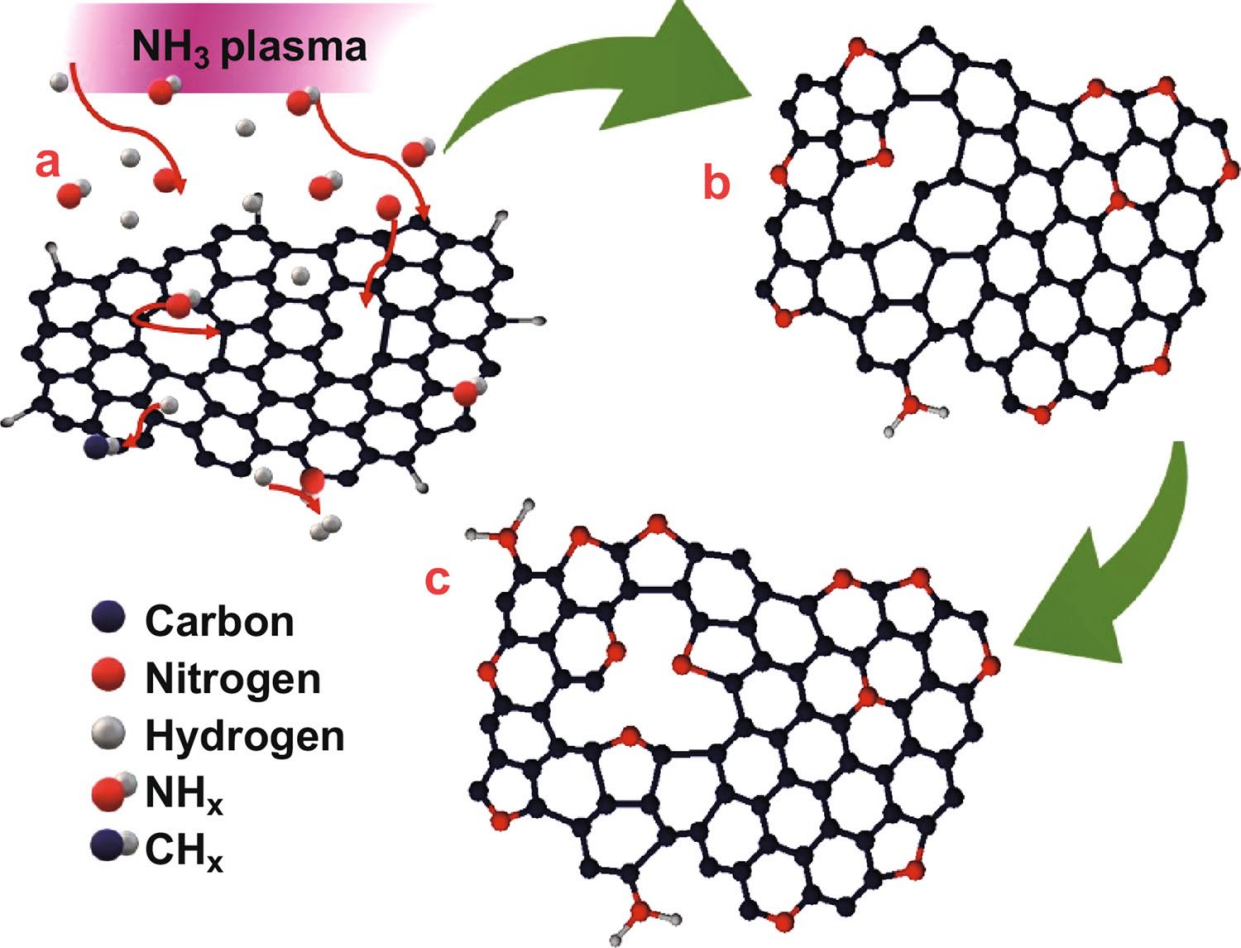
Schematic diagram of the plausible mechanism of nitrogen incorporation in CNWs using NH_3_ plasma post-treatment: **a** the interaction of plasma species with defects, **b** the substitution of N-atoms in the SV to form graphitic N and incorporating N at DV to form pyrrolic N, and **c** the transformation of pyrrolic N to form pyridinic N along with pyrrolic N at DV

Taking all these results into account suggests that building N-CNWs by plasma post-treatment has two stages: the plasma-enhanced defect generation and the incorporation of nitrogen into defects. Plasma-enhanced defect generation is mainly due to the removal of carbon atoms by the interaction of plasma species with the CNWs. The changes observed in the “fingerprint” region of NEXAFS spectra, the concentration of incorporated nitrogen and changes in the in-plane crystal size of N_2_ and NH_3_ plasma-treated samples are different, which indicates that the plasma-enhanced defect generation by the plasma species and nitrogen incorporation takes place differently. A typical NH_3_ plasma contains excited species (NH_3_* and N_2_*), different kinds of ions and radical species (NH_2_, NH and H)  [72], and N_2_ plasma contains excited N_2_ species, reactive N-atoms, N^2+^ and N^4+^ ions, which interact with H-terminated CNWs. The significant difference between NH_3_ and N_2_ plasma species is the presence of NH_*x*_ (*x* = 1, 2, 3) species and hydrogen species in the NH_3_ plasma (Fig. S8). NH_*x*_ (*x* = 1, 2, 3) species can chemically functionalise the edges of carbon to the formation of amino groups that are observed in the XPS spectra. The capability of hydrogen to etch the carbon resulted in the creation of more structural defects. The active N-containing species in NH_3_ plasma could react with the defects and incorporate nitrogen to the structure [73]. NEXAFS results propose that the defects formed during plasma treatment are DV (5-8-5) and SV (5-9). Transformation of DV (5-8-5) to stable DV (555-777) is well known [74, 75], can be made by the ion bombardment during the plasma exposure, which is the favourable site for nitrogen incorporation. Based on these findings, a plausible mechanism of nitrogen incorporation by NH_3_ plasma in three stages is summarised in Fig. 6: (a) the plasma-enhanced defect generation and interaction of plasma species with the defects; (b) the interaction of N-containing species at the SV defect site forms graphitic N, at DV defect forms pyrrolic N with an SV and at the edges forms amines, pyridinic N and pyrrolic N; and (c) the formation of pyridinic N by the transformation of pyrrolic N at DV after longer exposure. The increase in pyrrolic N in the graphene lattice decreases the in-plane crystalline size, which is evident from Raman.

**Fig. 7:**
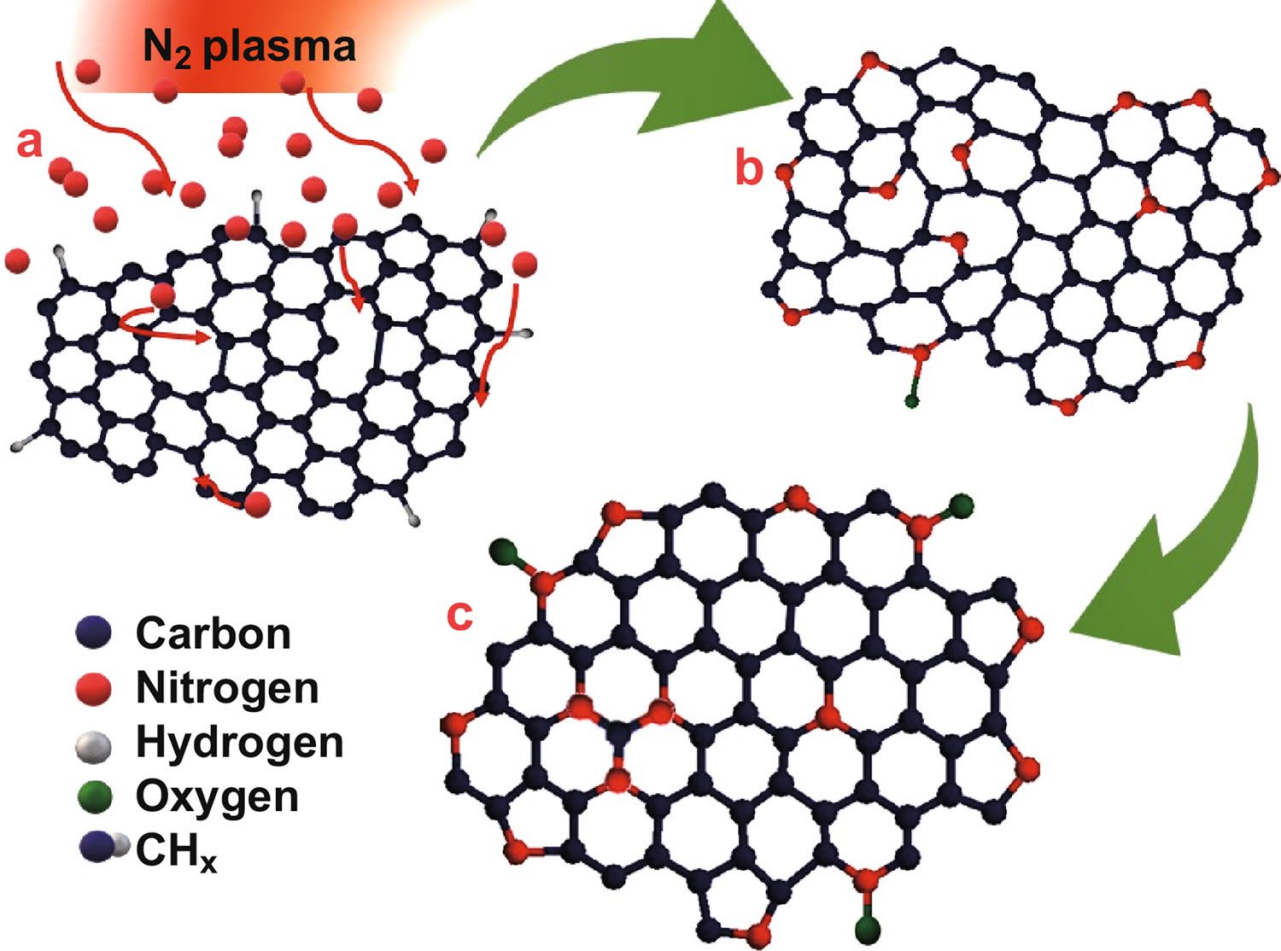
Schematic diagram of the possible mechanism of nitrogen incorporation in CNWs by N_2_ plasma post-treatment: **a** the interaction of plasma species with defects, **b** the substitution of N-atoms in the SV to form graphitic N and incorporating N at DV to form pyridinic N, and lastly **c** the transformation of pyridinic N to graphitic N by the bond rotation at DV and formation of oxidised pyridinic N groups by ex situ adsorption of oxygen

In the case of N_2_ plasma treatment, the nitrogen species interact with the H-terminated CNWs and form N-CNWs. There is no characteristic peak identified for SV defects in the NEXAFS spectrum after N_2_ plasma treatment, which confirms that only DV defects are formed during the plasma exposure. XPS analysis and Raman analysis exhibit the improvement in *sp*^2^-components with an increase in the in-plane crystalline size which indicates the reduction of vacancy defect by forming graphitic N, where the position of N-atom is almost the same as the C-atom in graphene [37]. Thus, the plausible mechanism of nitrogen incorporation by nitrogen plasma is summarised in Fig. 7 and comprised of three stages: (a) the plasma-enhanced defect generation and interaction of plasma species with the defects; (b) the interaction of N-containing species at the SV defect site forms graphitic N, at DV defect pyridinic N, and at edges pyridinic N, oxides of pyridinic N and pyrrolic N; and (c) the formation of more pyrrolic N at the edges and transformation of pyridinic N to graphitic N at DV sites by the defect migration [44, 48]. In both cases of plasma treatment, the structural defect generation in graphene and interaction of nitrogen with the structural defects play a key role in the formation of N-graphene/N-CNWs with different nitrogen configurations.

**Fig. 8:**
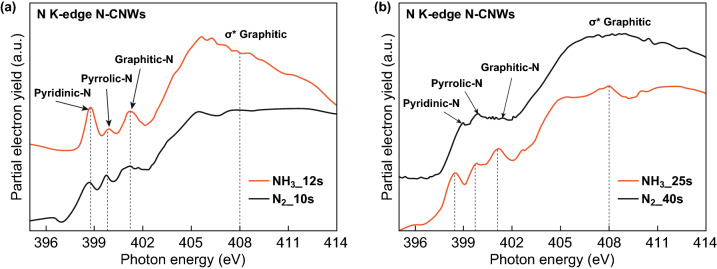
N K-edge spectra of the NH_3_ and N_2_ plasma-treated CNWs at different plasma exposure times

N K edges of N-CNWs after different plasma-treated conditions are observed and confirm the previously mentioned nitrogen incorporation mechanism. For the comparison, N K edge of 12 s NH_3_ plasma-treated CNWs and 10 s N_2_ plasma-treated CNWs is presented in Fig. 8a. The N K-edge spectra of N-CNWs after longer NH_3_ and N_2_ plasma post-treatment are shown in Fig. 8b. All of the samples possess three distinguished peaks in the π* region. The peak observed at ~ 398.5–399 eV falls into the pyridinic N behaviour of the N 1s core-level spectra. The appearance of a peak at ~ 399.8–400.2 eV is assigned to the presence of pyrrolic N in the structure. The peak observed at 401.0–401.5 eV agrees on the existence of graphitic N in the graphene structure. The evolution of peaks above 405 eV can be associated with the σ* resonance [40]. Observed results in the N K-edge spectra of N-CNWs have strong agreement with the features explained in the N 1s spectra (Fig. 4b, e). The peak intensity from graphitic N after 40 s N_2_ plasma-treated samples is decreasing while that for the prolonged NH_3_ plasma treatment remains almost similar. This indicates that the prolonged N_2_ plasma treatment leads to the formation of more pyrrolic N, which has a perfect agreement with the above-mentioned proposed nitrogen incorporation mechanism.

**Fig. 9:**
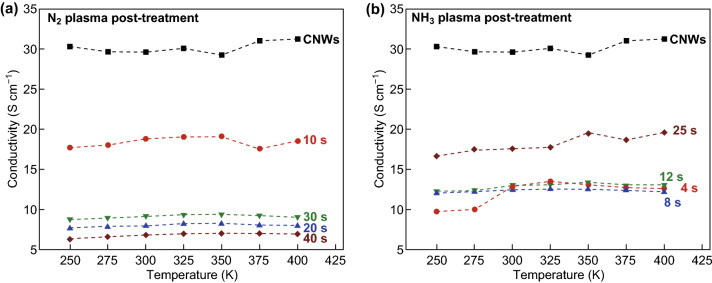
**a**, **b** Temperature-dependent electrical conductivity of CNW and N-CNWs: **a** after N_2_, and **b** after NH_3_ plasma treatment

Four-point van der Pauw measurement was carried out to study the effect of nitrogen incorporation in CNWs on the electrical conductivity of N-CNWs at different temperatures, which is represented in Fig. 9. The conductivity of CNWs decreases with the initial plasma treatment in both cases. Compared to NH_3_ plasma treatment, N-CNWs after N_2_ plasma treatment have minimum conductivity. For N_2_ plasma-treated CNWs, conductivity decreases with plasma exposure time. However, after NH_3_ plasma treatment, the conductivity falls initially and increases with longer plasma exposure time. This result strongly agrees with the earlier studies based on Kubo-Greenwood approach done by Lherbier et al. [25], which suggest that the conductivity is only marginally affected when the N concentration is between 2 and 4%, and when the concentration is higher than 5%, the electron mobility of N-graphene largely decreases. Therefore, a higher concentration of nitrogen after N_2_ plasma (~ 8%) leads to a decrease in the conductivity. In the case of NH_3_ plasma-treated samples, conductivity decreases initially with the plasma exposure and increases with the concentration of substituted nitrogen increasing. The significant presence of oxygenated groups after the plasma treatment also causes the reduced conductivity.

## Conclusions

Nitrogen-incorporated CNWs were successfully produced by the low-pressure RFICP system using N_2_ and NH_3_ cold-temperature plasma post-treatments. Mechanism of the incorporation and substitution of nitrogen in the CNWs and the influence of different plasma environments for controlling the concentration and configuration of nitrogen were successfully studied. The high concentrations of incorporated nitrogen ~ 8% after N_2_ plasma post-treatment and ~ 2.8% after NH_3_ plasma treatment give the possibility to control the nitrogen concentration using different plasma environments. Moreover, N_2_ plasma treatment produces N-CNWs with higher graphitic N concentration, while NH_3_ plasma treatment mainly produces N-CNWs with higher pyridinic N indicating the effective control of nitrogen configuration by choosing suitable plasma environments. Also, time-dependent plasma post-treatment studies give an insight into the change in the crystalline size of the nanowalls. Raman studies on the in-plane crystalline size explain that the crystalline order of graphene is improved by healing the vacancy defect by incorporating N-atom. Also, a better understanding of the defect generation and interaction of nitrogen with the bonding environments in nanowalls during the plasma post-treatment was obtained from NEXAFS analysis. All these experimental observations strongly corroborate the reported DFT calculations. Based on these findings, we propose a mechanism to explain the interaction of nitrogen with the defects in CNWs for the incorporation and substitution of nitrogen in the graphene lattice. The electrical conductivity measurements also strongly support other reported studies, where the conductivity decreases with the higher nitrogen concentration and increases with optimum graphitic N content. To this end, our studies provide a rapid, cost-efficient and environment-friendly technique for the synthesis of N-CNWs via plasma post-treatment processes at low pressure. This strong agreement of experimental results with the existing theoretical calculations on the nitrogen incorporation mechanism will set the base for the controlled synthesis of N-CNWs/N-graphene with desired nitrogen concentration and configurations by choosing optimum plasma parameters for future electronic applications.

## Electronic Supplementary Material

Below is the link to the electronic supplementary material.

Supplementary material 1 (PDF 797 kb)
