# Supplementary material for: N-Graphene Nanowalls via Plasma Nitrogen Incorporation and Substitution: The Experimental Evidence
Source: Nanomicro Lett. 2020 Feb 17;12:53. doi: 10.1007/s40820-020-0395-5 (PMC7770896; doi:10.1007/s40820-020-0395-5)
Supplement: Supplementary file 1 — Supplementary material 1 (PDF 797 kb) [file 40820_2020_395_MOESM1_ESM.pdf]

Supporting Information for

N-Graphene Nanowalls Via Plasma Nitrogen Incorporation and Substitution: The Experimental Evidence

Neelakandan M Santhosh[1, 2], Gregor Filipič [1], Eva Kovacevic[3], Andrea Jagodar[3], Johannes Berndt[3], Thomas Strunskus[4], Hiroki Kondo[5], Masaru Hori[5], Elena Tatarova[6], Uroš Cvelbar[1, *]

[1]Jožef Stefan Institute, Jamova cesta 39, SI-1000 Ljubljana, Slovenia, EU

[2]Jozef Stefan International Postgraduate School, Jamova cesta 39, SI-1000 Ljubljana, Slovenia, EU

[3]GREMI CNRS-University of Orleans, 14 rue d'Issoudun, 45067 Orleans Cedex 2, France

[4]Christian Albrechts University Kiel, Institute for Materials Science, Kaiserstr, 2, D-24143 Kiel, Germany

[5]Department of Electrical Engineering and Computer Science, University of Nagoya, Furo-cho Chikusa-ku, Aichi, Nagoya 464-8603, Japan

[6]Instituto de Plasmas e Fusão Nuclear, Instituto Superior Técnico, Universidade de Lisboa, Lisboa-1049, Portugal, EU

*Corresponding author. E-mail: uros.cvelbar@ijs.si (Uroš Cvelbar)

Supplementary Figures and Tables

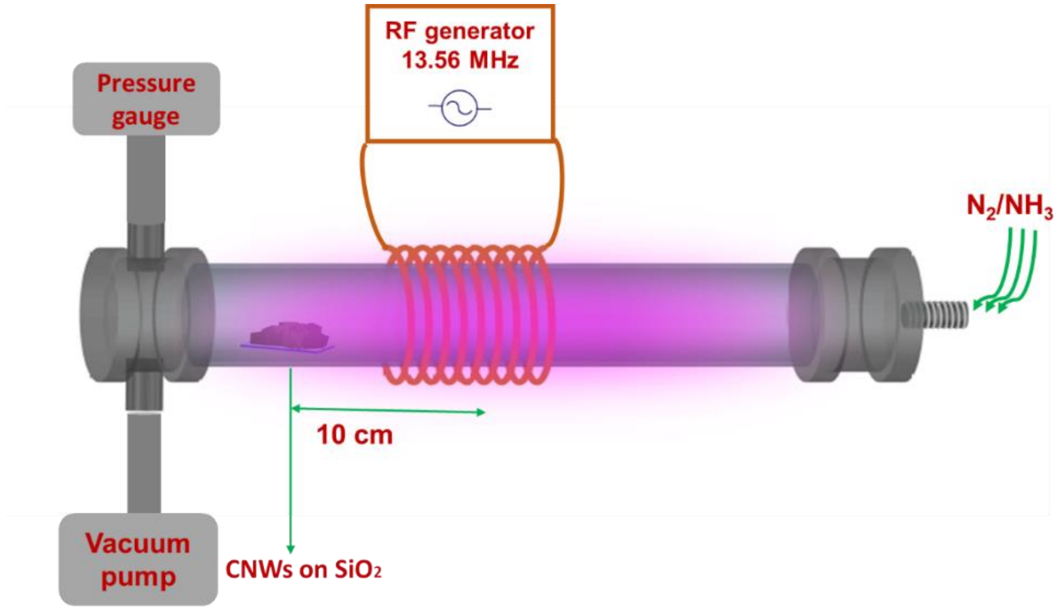

Fig. S1 Experimental setup of RFICP systems for producing N-CNWs

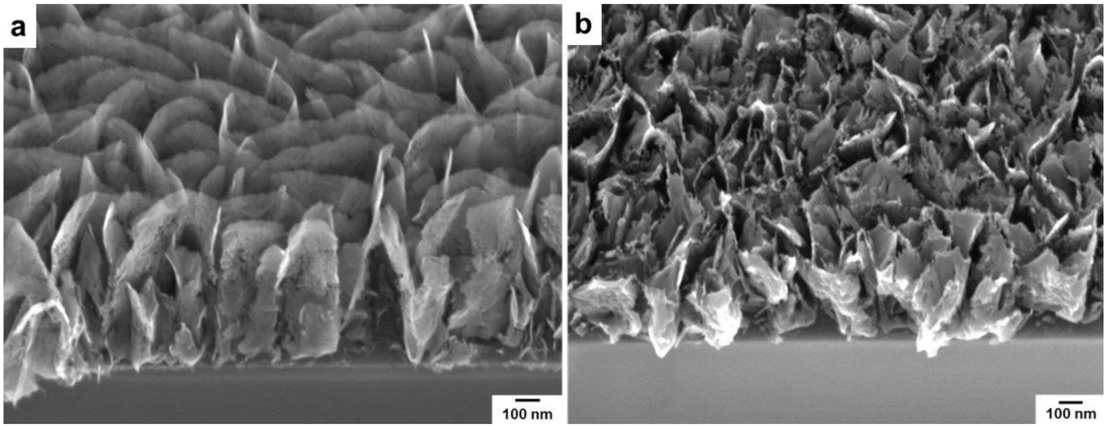

Fig. S2 a Tilted image of CNWs. **b** N-CNWs after 40s N_2 plasma exposure

Table S1 Peak position, I_D/I_G ratio, the in-plane crystallite size of CNWs and N-CNWs

**NH_3 plasma post-treatment**					**N_2 plasma post-treatment**				
**Sample conditions**	**D peak (cm^{-1})**	**G peak (cm^{-1})**	**I_D/I_G ratio**	**L_a (nm)**	**Sample conditions**	**D peak (cm^{-1})**	**G peak (cm^{-1})**	**I_D/I_G ratio**	**L_a (nm)**
0	1330	1586	2.85	13.53	0	1330	1586	2.85	13.53
4	1330	1586	2.78	13.84	10	1333	1587	2.72	14.17
8	1331	1585	2.71	14.21	20	1333	1587	2.59	14.88
12	1332	1584	2.68	14.36	30	1332	1585	2.48	15.54
25	1333	1587	2.98	12.93	40	1331	1585	2.28	16.90

Tuinstra-Koenig relationship to calculate in-plane crystallite size using I_D/I_G ratio:

$$L_a(nm) = 2.4 * 10^{-10} * \lambda^4 * (I_D/I_G)\text{-}1 \qquad (S1)$$

L_a is the in-plane crystallite size

λ is the Raman excitation wavelength (633 nm)

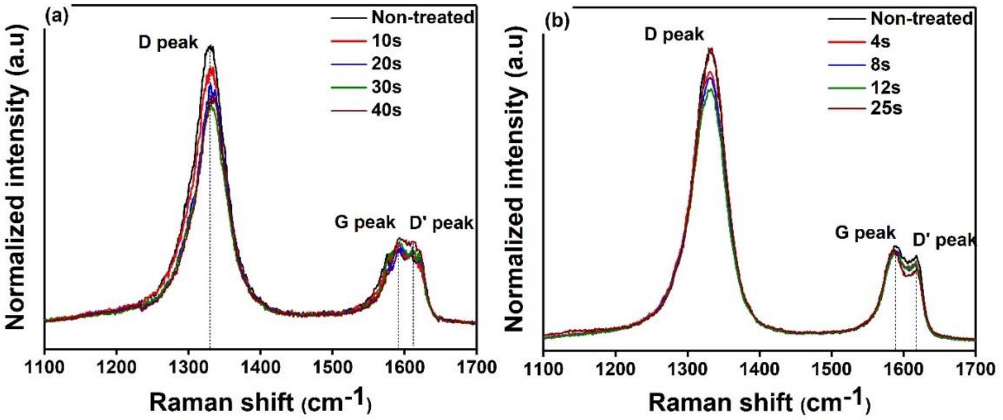

Fig. S3 Magnified region of D, G, and D’ peak after **a** N_2 plasma post-treatment. **b** NH_3 plasma post-treatment

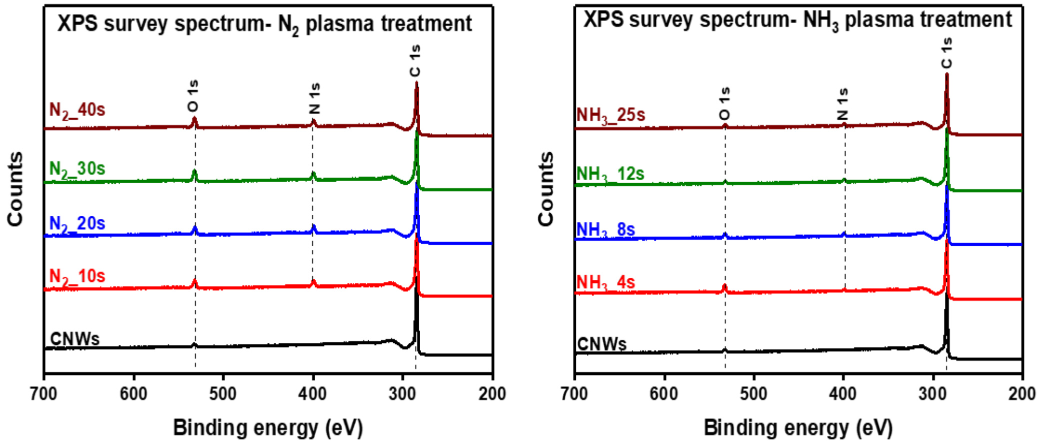

Fig. S4 XPS survey spectra of the samples before and after post-treatment. **a** N_2 plasma. **b** NH_3 plasma

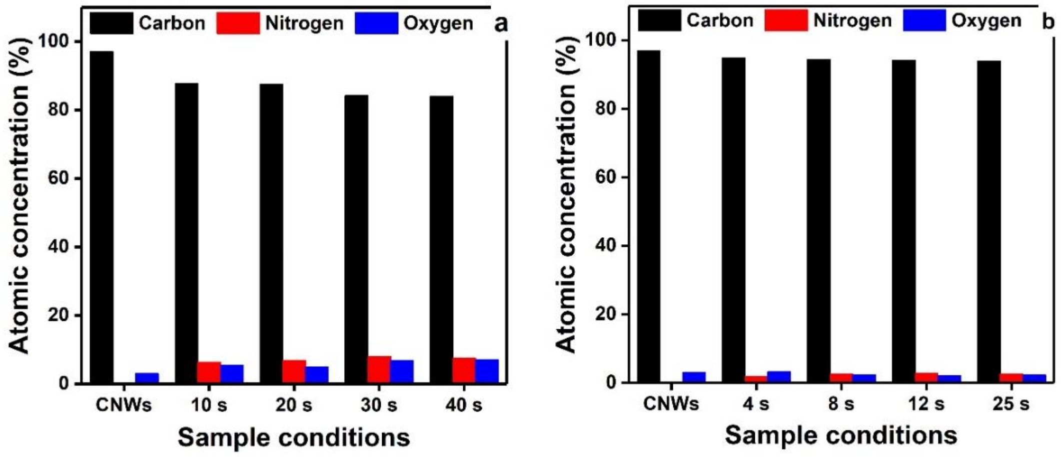

Fig. S5 The estimated atomic concentration of carbon, nitrogen, and oxygen after plasma treatment. **a** N_2 plasma. **b** NH_3 plasma

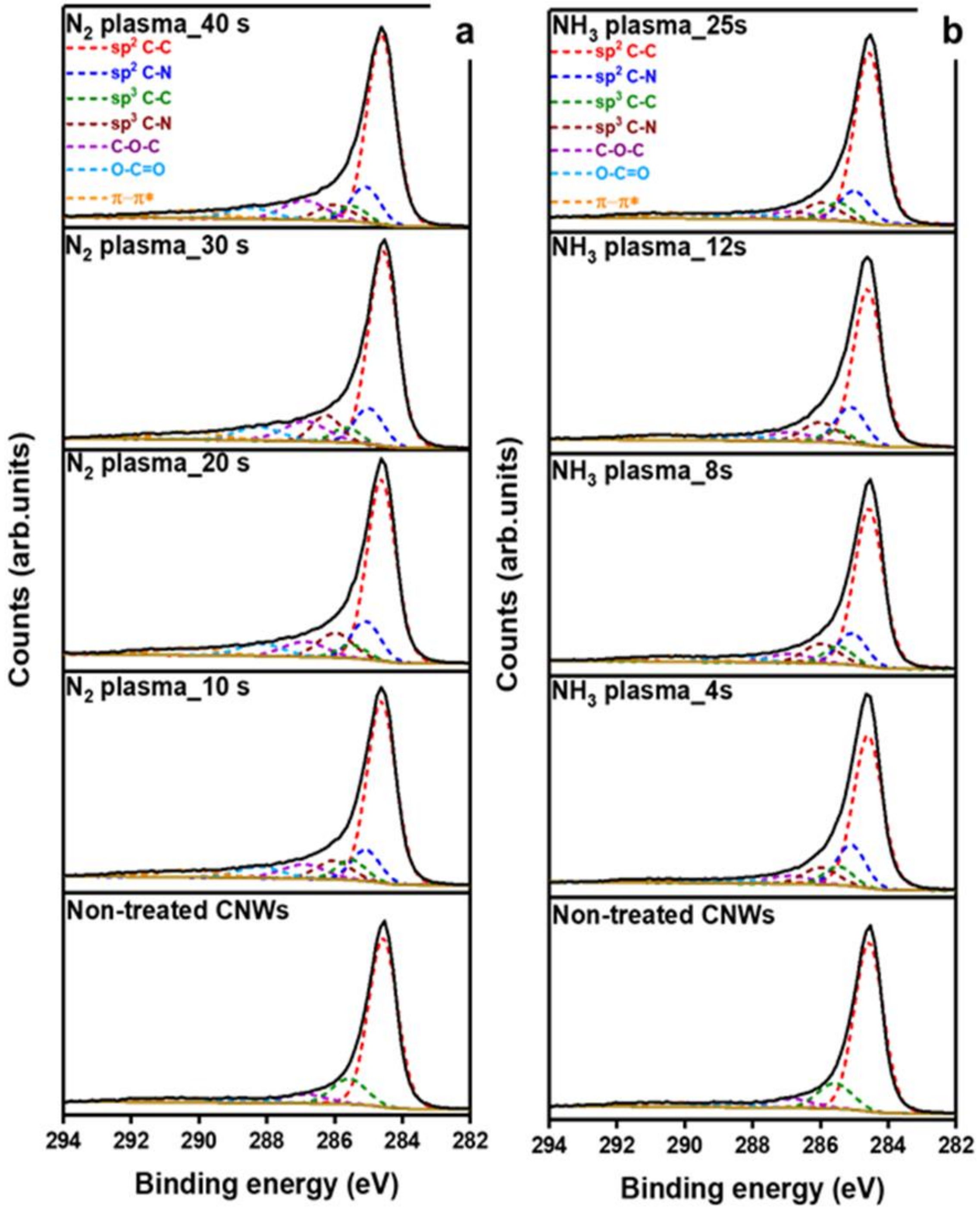

Fig. S6 Detailed C 1s region of the samples before and after **a** N_2 plasma post-treatment and **b** NH_3 plasma-treatment.

Table S2 Peak position, FWHM and roughly estimated concentration of the peak components in C 1s after N_2 plasma treatment

Peak position	CNWs		10 s		20 s		30 s		40 s	
	FWHM (eV)	Area %	FWHM (eV)	Area %	FWHM (eV)	Area %	FWHM (eV)	Area %	FWHM (eV)	Area %
284.6 eV (sp^2 C-C)	1	74	1	66.5	1	62.5	1	61.5	1	59.4
285.1 eV (sp^2 C-N)	1		1	7.2	1	11.4	1	13.2	1	15.7
285.5 eV (sp^3 C-C)	1.2	12.7	1.2	7.0	1.2	4.3	1.2	4.3	1.2	4.9
286.2 eV (sp^3 C-N)	1.2		1.2	5.6	1.2	8.5	1.2	8.5	1.2	6.1
286.9 eV (C-O-C)	1.5	6.1	1.5	5.8	1.5	4.7	1.5	4.7	1.5	4.7
288.2 eV (O-C=O)	1.5	2.8	1.5	4.8	1.5	3.6	1.5	2.8	1.5	4.8
290.3 eV (π-π*)	2	4.2	2	3.1	2	4.6	2	4.6	2	4.0

Table S3 Peak position, FWHM and roughly estimated concentration of the peak components in C 1s after NH_3 plasma treatment

Peak position	CNWs		4 s		8 s		12 s		25 s	
	FWHM (eV)	Area %	FWHM (eV)	Area %	FWHM (eV)	Area %	FWHM (eV)	Area %	FWHM (eV)	Area %
284.6 eV (sp^2 C-C)	1	74.0	1	59.9	1	59.9	1	58.7	1	58.8
285.1 eV (sp^2 C-N)	1		1	8.9	1	8.0	1	9.8	1	8.4
285.5 eV (sp^3 C-C)	1.2	12.7	1.2	7.6	1.2	8.2	1.2	7.6	1.2	9.9
286.2 eV (sp^3 C-N)	1.2		1.2	4.4	1.2	9.2	1.2	7.0	1.2	9.7
286.9 eV (C-O-C)	1.5	6.1	1.5	7.9	1.5	7.1	1.5	7.2	1.5	4.4
288.2 eV (O-C=O)	1.5	2.8	1.5	5.9	1.5	6.1	1.5	4.1	1.5	4.3
290.3 eV (π-π*)	2.0	4.2	2.0	5.0	2.0	5.1	2.0	5.3	2.0	4.4

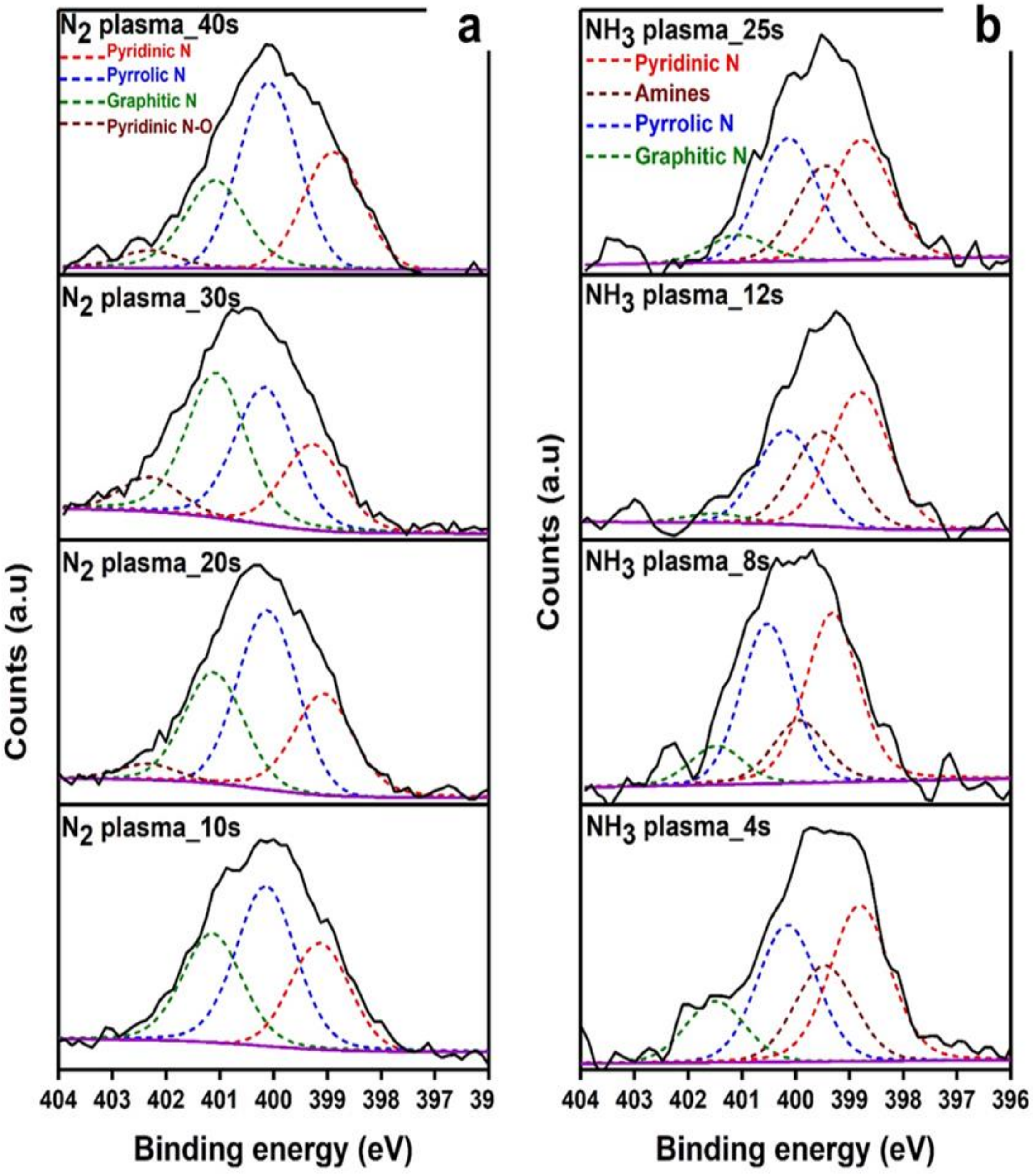

Fig. S7 Detailed XPS N 1s region of the samples before and after **a** N_2 plasma-treatment and **b** NH_3 plasma-treatment

Table S4 Peak position, FWHM and roughly estimated concentration of the peak components of N 1s after N_2 plasma treatment

Sample condition	Pyridinic N			Pyrrolic N			Graphitic N			Pyridinic N-O		
	Position (eV)	FWHM (eV)	Area %	Position (eV)	FWHM (eV)	Area %	Position (eV)	FWHM (eV)	Area %	Position (eV)	FWHM (eV)	Area %
10s	398.9	1.3	26.5	400.1	1.3	43.7	401.1	1.3	29.8			
20s	399.1	1.3	27.1	400.1	1.3	41.8	401.1	1.3	27.1	402.4	1.3	4.0
30s	399.3	1.3	21.9	400.2	1.3	33.5	401.1	1.3	36.9	402.4	1.3	8.4
40s	398.9	1.3	27.7	400.1	1.3	43.7	401.1	1.3	23.7	402.4	1.3	4.9

Table S5 Peak position, FWHM and roughly estimated concentration of the peak components of N 1s after NH_3 plasma treatment

Sample condition	Pyridinic N			Amines			Pyrrolic N			Graphitic N		
	Position (eV)	FWHM (eV)	Area %	Position (eV)	FWHM (eV)	Area %	Position (eV)	FWHM (eV)	Area %	Position (eV)	FWHM (eV)	Area %
4s	398.8	1.3	35.5	399.5	1.3	21.5	400.1	1.3	28.8	401.2	1.3	14.0
8s	398.8	1.3	40.7	399.5	1.3	15.1	400.2	1.3	35.2	401.3	1.3	8.9
12s	398.8	1.3	41.3	399.5	1.3	28.6	400.2	1.3	26.1	401.3	1.3	3.8
25s	398.8	1.3	30.4	399.5	1.3	26.3	400.2	1.3	32.6	401.2	1.3	11.8

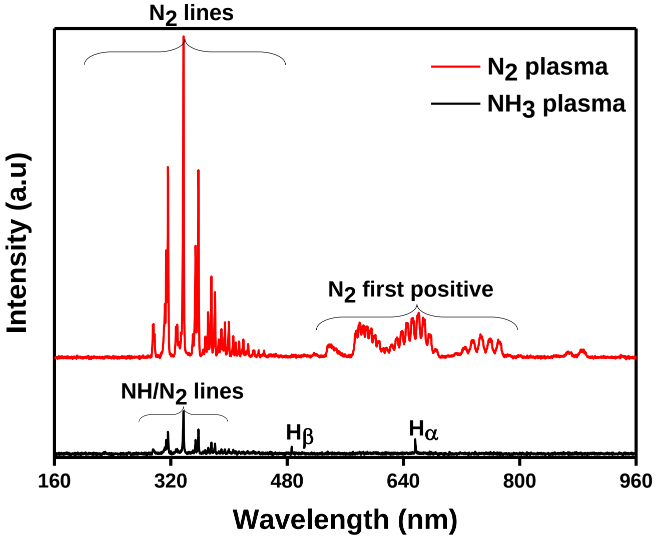

Fig. S8 The optical emission spectrum of the N_2 and NH_3 plasma in the RFICP system for an RF power of 300W
